# Aum Shinrikyo: once and future threat?

**DOI:** 10.3201/eid0504.990409

**Published:** 1999

**Authors:** K B Olson

**Affiliations:** Research Planning, Inc., Arlington, Virginia 22042, USA. kolson@rpihq.com


### Aum Shinrikyo: Once and Future Threat?

**Kyle B. Olson**
Research Planning, Inc., Arlington, Virginia, USA

On March 20, 1995, members of the Aum Shinrikyo cult entered the Tokyo subway system and released sarin, a deadly nerve agent. The subway attack was the most deadly assault in an ongoing campaign of terror waged by this mysterious cult. Four years later, with Aum Shinrikyo attempting to rebuild itself, many in Japan and around the world are asking whether the "Supreme Truth Sect" poses a current or future threat. Answering this question may further our understanding, not only of the Aum but also of other extremist and terrorist groups.

Aum Shinrikyo began its public campaign of terror on June 27, 1994. On that Monday in Matsumoto, a city of 300,000 population 322 kilometers northwest of Tokyo, a group of cult members drove a converted refrigerator truck into a nondescript residential neighborhood. Parking in a secluded parking lot behind a stand of trees, they activated a computer-controlled system to release a cloud of sarin. The nerve agent floated toward a cluster of private homes, a mid-rise apartment building, town homes, and a small dormitory.

This neighborhood was targeted for a specific reason. The dormitory was the residence of all three judges sitting on a panel hearing a lawsuit over a real-estate dispute in which Aum Shinrikyo was the defendant. Cult lawyers had advised the sect's leadership that the decision was likely to go against them. Unwilling to accept a costly reversal, Aum responded by sending a team to Matsumoto to guarantee that the judges did not hand down an adverse judgment. A light breeze (3 to 5 knots) gently pushed the deadly aerosol cloud of sarin into a courtyard formed by the buildings. The deadly agent affected the inhabitants of many of the buildings, entering through windows and doorways, left open to the warm night air. Within a short time, seven people were dead. Five hundred others were transported to local hospitals, where approximately 200 would require at least one night's hospitalization.

After successfully completing their mission, the cultists drove off to Kamakuishki, a rural community at the foot of Mount Fuji, home to golf courses, parks, dairy farms, small villages, and the headquarters of Aum Shinrikyo in Japan. The cult's facilities consisted of a number of motley buildings, factories, and dormitories.

Aum Shinrikyo's next major act of violence would serve as a wake-up call to the world regarding the prospects of weapons of mass destruction and terrorism. On the morning of March 20, 1995, packages were placed on five different trains in the Tokyo subway system. The packages consisted of plastic bags filled with a chemical mix and wrapped inside newspapers. Once placed on the floor of the subway car, each bag was punctured with a sharpened umbrella tip, and the material was allowed to spill onto the floor of the subway car. As the liquid spread out and evaporated, vaporous agent spread throughout the car.

Tokyo was experiencing a coordinated, simultaneous, multi-point assault. The attack was carried out at virtually the same moment at five different locations in the world's largest city: five trains, many kilometers apart, all converging on the center of Tokyo. The resulting deaths and injuries were spread throughout central Tokyo. First reports came from the inner suburbs and then, very quickly, cries for help began to flow in from one station after another, forming a rapidly tightening ring around the station at Kasumagaseki. This station serves the buildings that house most of the key agencies of the Japanese government. Most of the major ministries, as well as the national police agency, have their headquarters at Kasumagaseki.

By the end of that day, 15 subway stations in the world's busiest subway system had been affected. Of these, stations along the Hbiya line were the most heavily affected, some with as many as 300 to 400 persons involved. The

Address for correspondence: Kyle B. Olson, Research Planning, Inc., 6400 Arlington Blvd., Suite 1100, Arlington, VA 22042, USA; fax: 703-237-8085; e-mail: kolson@rpihq.com.

number injured in the attacks was just under 3,800. Of those, nearly 1,000 actually required hospitalization—some for no more than a few hours, some for many days. A very few are still hospitalized. And 12 people were dead.

Within 48 hours of the subway attack, police were carrying out raids against Aum Shinrikyo facilities throughout Japan. Police entered cult facilities carrying sophisticated detection systems and wearing military-issued chemical gear (which was issued to the Tokyo police the week before the subway attack).

The real target of the raids that began on March 17 was the building known as Satyan 7, a supposed shrine to the Hindu god Shiva, the most prominent figure in the Aum Shinrikyo religious pantheon. In reality, the building housed a moderately large-scale chemical weapons production facility, designed by cult engineers, with first-rate equipment purchased over-the-counter.

Although the facility's design was crude by industry standards, it was nonetheless very capable of producing the sarin used in the Matsumoto attack. At the time of the Tokyo attack, however, Satyan 7 was not in service, having been mothballed after an accident during the previous summer. In an effort to get the plant back into production, the cult had, during the fall of 1994, unsuccessfully attempted to recruit Russian chemical-weapons engineers. The cult was adept at recruiting educated professionals (scientists and engineers), but most were young and largely inexperienced. Satyan 7 was designed to produce sarin, not on a small terrorist scale, but in nearly battlefield quantities: thousands of kilograms a year.

Chemical weapons were not, however, the only option available to the Aum. The first cult laboratory for toxin production was actually in place by 1990 and was subsequently replaced with two new laboratories, one at Kamakuishki and the other in Tokyo. Aum dabbled in many different biological agents. They cultured and experimented with botulin toxin, anthrax, cholera, and Q fever. In 1993, Ashahara led a group of 16 cult doctors and nurses to Zaire, on a supposed medical mission. The actual purpose of the trip to Central Africa was to learn as much as possible about and, ideally, to bring back samples of Ebola virus. In early 1994, cult doctors were quoted on Russian radio as discussing the possibility of using Ebola as a biological weapon.

The cult attempted several apparently unsuccessful acts of biological terrorism in Japan between 1990 and 1995. As early as April 1990, the cult had tried to release botulin toxin from a vehicle driving around the Diet and other government buildings in central Tokyo. In early June of 1993, another attempt was made to release botulin toxin, this time in conjunction with the wedding of the crown prince. A vehicle equipped with a spray device was driven around the imperial palace as well as the main government buildings in central Tokyo.

Later that month, pursuing an alternative technology, the cult attempted to release anthrax spores from its mid-rise Tokyo office building laboratory. At that time, police and media reported foul smells, brown steam, some pet deaths, and stains on cars and sidewalks. Then, in March 1995, just before the sarin subway attack, an attempt to spray botulin toxin in the subway at Kasumagaseki Station was preempted by a cult member who opted not to load the improvised briefcase sprayers with actual agent.

No injuries were reported in any of these biological events despite the fact the cult was dealing with very toxic materials. The cult's failures can be attributed to a variety of factors. The cult may not have had the right agents or the right technologic facilities; they could have overcooked the bioagents or not known how to use them. While the cult was well financed, it was not very successful in its efforts to recruit biological scientists. Still, the possibility exists that casualties associated with some of these releases might have not been detected or were attributed to other causes.

The cult's operations were worldwide, promoting a theology drawn from different sources, including Buddhism, Christianity, Shamanism, Hinduism, and New Age beliefs. Cult membership around the world was likely 20,000 to 40,000. One cult leader estimated the cult's net worth in March of 1995 at about $1.5 billion. The money was collected through donations, tithing, sales of religious paraphernalia, videotape and book sales, and other sources. The cult conducted seminars and hosted training courses for members, offering indoctrination in Aum's teachings, charging believers from hundreds to tens of thousands of dollars for attending these sessions. Aum Shinrikyo also had a number of commercial enterprises, even a company that manufactured computers. Imported components from Taiwan were assembled in a cult factory at Kamakuishki and sold in Aum's computer store in downtown Tokyo. The cult also ran a chain of restaurants in Tokyo and several other Japanese cities.

Another source of income was the practice of green mail. Aum would threaten to establish a cult compound in a city and, if the city fathers did not bribe them to go away, the cult would set up shop. Several cities paid rather than have Aum establish operations there. The cult manufactured illegal drugs and had a marketing agreement with the Japanese Mafia (the Yakuza). In 1996, the Yakuza would be found responsible for the assassination of the cult's lead scientist, Dr. Hideo Murai, in the days following the Tokyo subway attack. Concerned at his frequent televised appearances, the Yakuza silenced him for fear that he would betray the linkage between the two shadowy groups. Extortion, theft, and murder were also part of the cult's fund-raising activities. Among the cult leaders, "Doomsday guru" Shoko Ashahara is the undisputed head. Ashahara (born Chizuo Matsumoto) had numerous exalted titles, including venerated master, yogi, and holy pope. Highly charismatic, this partially blind, apparently very talented yoga instructor was very ambitious politically and financially. He and more than 20 of his followers ran for Parliament in 1989. They were defeated, which some Japanese analysts have suggested marks the moment when the cult's leader elected to pursue weapons of mass destruction and the violent overthrow of the established order.

Millennial visions and apocalyptic scenarios dominate the group's doctrine, evidenced by the prominent role of Nostradamus as a prophet in Aum Shinrikyo teaching. Ashahara has, on many occasions, claimed to be the reincarnated Jesus Christ, as well as the first "enlightened one" since the Buddha. He has frequently preached about a coming Armageddon, which he describes as a global conflict that would, among other things, destroy Japan with nuclear, biological, and chemical weapons. According to Ashahara, only the followers of Aum Shinrikyo will survive this conflagration.

Another cult leader, Fumihiro Joyu, now 35 years old, was a bright young engineer with the Japanese space program, specializing in artificial intelligence. He left that organization to go to work for Aum, where he very quickly rose through the ranks, ultimately to head the cult's operations in Russia. Joyu oversaw this important cult expansion, among other things "investing" as much as $12 million in the form of payoffs to well-placed officials. The cult's investment paid off with expedited access to office buildings, dormitories, and other facilities throughout Russia. At the time of the Tokyo subway attack, the cult's principle venture in Russia was the Moscow-Japan University, with headquarters in offices across the street from the Bolshoi Ballet. Their senior Russian partner in the university was a man by the name of Oleg Lobov, at that time also chairman of Russia's National Security Council and a close confidant of Boris Yeltsin.

Joyu was convicted of perjury after the subway investigation, but he received an extremely light sentence (3 years) for his involvement in the cult's activities. Joyu has apparently maintained close ties to the cult, and he is slated for release toward the end of this year. After leaving prison, he may make a play for leadership of the remaining cult elements. He is the most charismatic member of the cult, other than Ashahara. In the days right after the Tokyo subway attack, he was on Japanese television so frequently, and featured in magazines and newspapers so often, that he became a teen heartthrob.

In the days and weeks immediately following the gas attack, more than 200 key members of the cult were arrested. Approximately 120 are still in jail, on trial, or have been convicted. Ashahara himself has been on trial for 3 years. The trial may continue for 5 or 6 years, a judicial timetable that is aggressive by Japanese standards in cases where the defendant refuses to cooperate with the prosecution. Three cult members involved in the attack are still at large. Russian operations were ended by legal action and the assets seized by the government. The cult's legal status in Japan as a church has been revoked, but many of its assets are unaccounted for.

Today, Aum Shinrikyo is once again soliciting donations, collecting tithes, selling materials to members, holding seminars, conducting training, and selling computers. Active recruiting is under way. Aum Shinrikyo is holding 50 "educational" seminars a month for current and potential members. The cult has offices throughout Japan, around Tokyo and other cities, and, according to Japanese sources, they maintain 100 hide-outs throughout that country as “safe houses.” These sources estimate that at least 700 members are live-in, fully committed devotees. Mind control is still a part of the cult’s package. Cult members can be seen in Aum-owned houses wearing bizarre electric headsets, supposedly designed to synchronize their brain waves with those of the cult’s leader.

What is the message that these events to impart policy-makers? The objective of the Tokyo subway attack was not irrational. The objective that day was to kill as many policemen as possible; Aum Shinrikyo had become aware of police plans to conduct raids against cult facilities, beginning on March 20. The cult’s timetable could not permit that interruption.

Aum’s actions were perfectly logical within the context of their value system. They were a self-legitimized group that had rejected and, ultimately, felt obliged to confront society. Outnumbered as they were by Japanese police and military might, one can argue that developing and even using an asymmetric capability was a logical consequence of their situation. Unable to achieve their objective—political power—through legitimate means, they determined that a preemptive strike was necessary.

Is Aum Shinrikyo a potential threat? Is Shoko Ashahara just the first of many, or has he been relegated to the scrap heap? These are open questions we will be forced to grapple with for many years to come.

Mr. Olson is adviser, consultant, and writer on high-technology terrorism, the threat of chemical and biological weapons, and the practical challenges of arms control; member of the Central Intelligence’s Nonproliferation Advisory Panel; guest lecturer on chemical and biological weapons terrorism at the Defense Nuclear Weapons School, Air War College, Naval War College, and U.S. Air Force Special Operations School, and an adjunct faculty member at George Washington University.

